# Design and Optimization of High-Power and Low-Frequency Broadband Transducer with Giant Magnetostrictive Material

**DOI:** 10.3390/s23010108

**Published:** 2022-12-22

**Authors:** Long Yang, Wenjie Wang, Xu Zhao, Haojun Li, Yue Xiang

**Affiliations:** School of Aerospace Engineering, Beijing Institute of Technology, Beijing 100081, China

**Keywords:** aerospace sensing system, giant magnetostrictive material, honeycomb structure, finite element simulation, harmonic response and harmonic acoustics simulation

## Abstract

The applications of sensors in the aerospace industry are mostly concentrated in the middle and high frequencies, and low-frequency sensors often face the problems of low power and short working bandwidth. A lightweight, thin, high-power, low-frequency broadband transducer based on giant magnetostrictive material is designed. The design and optimization processes of the core components are introduced and analyzed emphatically. The finite element simulation results are validated by the PSV-100 laser vibration meter. Three basic configurations of the work panel are proposed, and the optimal configuration is determined by modal, acoustic, and vibration coupling analyses. Compared with the original configuration, it is found that the lowest resonant frequency of the optimal configuration is reduced by 24.6% and the highest resonant frequency within 2000 Hz is 1744.9 Hz, which is 54.2% higher than that of the original configuration. This greatly improves the vibration power and operating frequency range of the transducer. Then, the honeycomb structure is innovatively applied to the work panel, and it is verified that the honeycomb structure has a great effect on the vibration performance of the work panel. By optimizing the size of the honeycomb structure, it is determined that the honeycomb structure can improve the vibration power of the work panel to its maximum value when the distance between the half-opposite sides of the hexagon is H = 3.5 mm. It can reduce the resonant frequency of the work panel; the lowest resonant frequency is reduced by 12.8%. At the same time, the application of a honeycomb panel structure can reduce the weight of the transducer.

## 1. Introduction

As a new type of material that can sense environmental stimuli and take certain measures to respond appropriately, smart materials are used to replace traditional metal materials. Smart materials are likely to dominate certain applications in the near future [1,2].

Piezoelectric ceramic materials are currently the most commonly used smart materials [3,4,5] in the field of aerospace engineering. The piezoelectric transducers are used in active vibration and noise suppression sensors for plate structures [6], vibration energy collection sensors [7], high-precision digital torque wrenches [8], mechanical strain measurement sensors [9], structural health monitoring (SHM) [10,11], and flaw detectors [12,13] et al. Piezoelectric is suitable for small sensors and high output power [14]. However, sometimes, some performance flaws of piezoelectric ceramic materials, such as a small size range, low frequency that is difficult to achieve [15], high voltage intolerance, high driving voltage [16], and material fading polarization [17], limit its application in the sensing system to a certain extent.

The new way to avoid those problems is the giant magnetostrictive material (GMM), which can replace the piezoelectric ceramic material. GMM is another important new smart material after rare earth permanent magnets, rare earth magneto-optical, and rare earth high temperature superconducting materials. It can effectively realize electric-magneto-mechanical energy conversion and has the advantages of a large magnetostrotive coefficient, high power capacity, and fast response speed [18]. The sensor based on the GMM has the characteristics of a high Curie temperature, a high magneto-mechanical coupling coefficient, a good frequency response, a large magnetostrictive strain value, and a large operating frequency bandwidth [19,20]. It is an attractive substitute for piezoelectric ceramic materials, and the giant magnetostrictive sensor has attracted much attention [21]. Ghorbanpour Arani et al. [22] studied the free vibration of magnetostrictive sandwich composite microplates with magnetostrictive cores and composite panels and found that the angle between the magnetic field and the composite fiber plays a key role in the dimensionless frequency of microplates. This can be used in the design of sensors and provide ideas for the vibration response control of systems in the aerospace industry. Backman et al. [23] designed a magnetostrictive cantilever sensor that could collect vibration energy and predicted the vibration frequencies of different cantilever configurations through the finite element model (FEM). They found that changing the cantilever design to a “T” shape could reduce the resonance frequency and provide an effective alternative for the power supply of a micro electro mechanical system (MEMS) in the aerospace engineering industry. Toutsop et al. [24] found the directional influence of the magnetic incremental permeability (MIP) sensor and proposed the directional magnetic incremental permeability. They found that the magnetic incremental permeability is related to the magnetization mechanism of the magnetic domain wall swelling. This provides ideas for improving the efficiency of electric energy conversion in the aviation field and is conducive to the weight reduction of related instruments. In their research, Liu et al. [25] found that the high-precision displacement sensor used to measure absolute displacement optimized the system structure and signal processing method. Then, they improved the bias magnetic field to achieve better energy exchange efficiency and used a copper ring to precisely control the dynamic magnetic field range to enhance the signal amplitude. The cross correlation algorithm was used to determine the delay time difference of the echo signal to determine the displacement value. Xie et al. [26] proposed a thin film magnetostrictive patch transducer (MPT), which can generate and receive single-mode and almost non-dispersive guided waves (GW), and has advantages such as light weight, flexibility, and high energy exchange efficiency. It can be used in SHM technology to detect and monitor curved thin-wall structures in the aerospace industry, so as to avoid life and economic losses. Leong et al. [27] researched the efficacy of magnetostrictive belt sensors as impact damage detectors for aerospace composites, and used tensile and three-point bending tests to study the sensitivity of embedded magnetostrictive belts and surface installation so as to promote structural flaw detection of composite materials through wireless sensing.

The application of GMM in the aerospace industry focuses on the middle and high frequencies [28], and transducers applied in the low frequency range often face problems such as low output power and short working bandwidth [29]. In this paper, a new thin low-frequency broadband transducer based on GMM is designed and is used to meet the requirements of high power, low working frequency, large broadband, high efficiency, and lightweight in the aerospace industry. It can improve the performance of aerospace sensors in the low-frequency range. The transducer designed in this paper uses the hinge structure to convert the hysteretic deformation into the longitudinal deformation of the work panel and reduce the longitudinal size of the transducer. The vibration mode of the transducer was simulated based on the FEM, and the accuracy of the finite element simulation was verified by the PSV-100 laser vibration meter. Three basic configurations of the work panel are proposed, and by comparing their resonant frequencies and vibration characteristics in the range of 0~2000 Hz, the optimal configuration is determined as the work panel of the transducer. In order to improve the vibration power of the work panel, the work panel is innovatively improved into the honeycomb panel structure, and the size parameters of the honeycomb structure are optimized to obtain the optimal honeycomb configuration. This improves the vibration power of the transducer and reduces the vibration frequency of the work panel, which provides a new idea for improving the performance of the sensor in the low frequency range.

## 2. Original Configuration of GMM Transducer

### 2.1. Basic Structure and Principle

**Figure 1:**
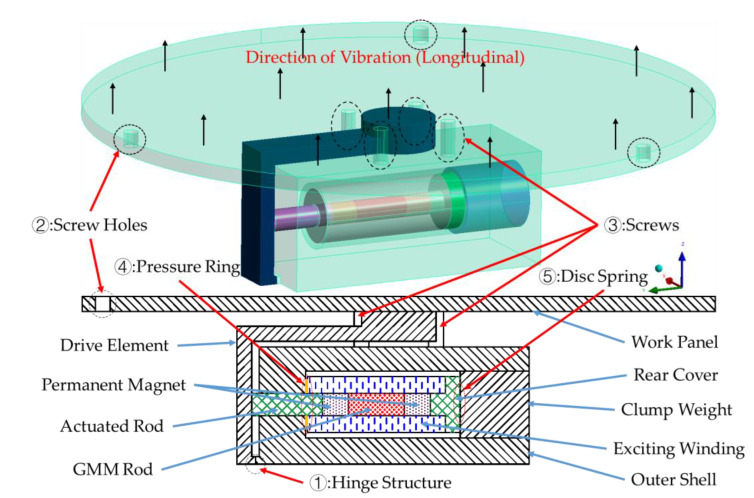
Original configuration diagram of the GMM transducer.

The simplified structure principle of the transducer is shown in Figure 1, and the components, such as the GMM rod, are marked with colored shadows due to their different materials. The transducer is of horizontal construction to reduce the longitudinal size, while the work panel is connected to the outer shell by four screws (as shown in Figure 1) to prevent the vibration of the work panel from separating from the other parts.

Due to the electromagnetic induction effect, when the current in the exciting winding changes, a magnetic field will be generated, and the GMM rod will have a large axial deformation under the action of the magnetic field [30]. In order to reduce the longitudinal size of the transducer, the drive element is used to convert the axial deformation into longitudinal deformation through the hinge structure in Figure 1, and the surface of the work panel is driven to generate vibration, so as to realize the mutual conversion of electromagnetic energy and mechanical energy. According to the working principle of the GMM transducer and the main factors affecting its performance, it can be divided into an excitation system, a magnetic bias system, a prestress system, and so on.

The prestress system is composed of a clump weight, a disc spring, a pressure ring, and screws. The disc spring is installed between the rear cover and clump weight, and its size and space occupied can be neglected, so only the installation location is marked in Figure 1. The pressure ring is set on the actuated rod and placed between the exciting winding and the outer shell, which can not only apply the prestress, but also prevent the transducer from entering the debris during the working process and affecting the performance of the exciting winding. At the same time, screws are used to fix the relative position between the clump weight and the outer shell to prevent it from moving. By applying axial prestressing force, the internal magnetic domain of the GMM bar is perpendicular to the axial stress when there is no excitation magnetic field. In addition, the multi-field coupling coefficient and the electromechanical coupling coefficient are also affected by the prestress. The conversion efficiency between mechanical energy and electromagnetic energy can be significantly improved by adjusting the appropriate prestress.

In the magnetic bias system, the permanent magnet can suppress the “frequency doubling” characteristic of GMM by generating a constant biased magnetic field [31] and change the initial working state of the GMM rod.

The excitation system changes the intensity of the magnetic field by adjusting the current in the exciting winding so that the GMM rod can output the required force and displacement to meet the operational requirements on specific occasions. The exciting winding is easy to generate heat, especially in the high frequency environment, which affects the working condition of the transducer. Therefore, it is usually necessary to develop the cooling system. The transducer designed in this paper is mainly used in the low-frequency environment and has its own cooling effect in the low-temperature environment at a high altitude, so there is no need for the design of the cooling system. The practical application of the transducer is the longitudinal vibration through the work panel for energy conversion, so the work panel must be fixed through the screw holes in Figure 1.

### 2.2. Structural Parameter Design

#### 2.2.1. Excitation Circuit Design

**Figure 2:**
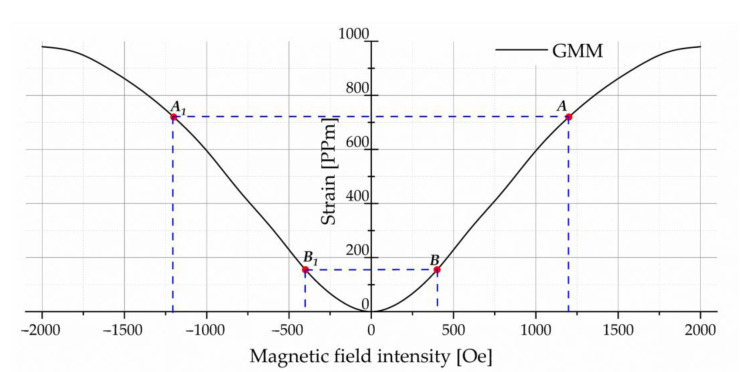
Relationship between the strain of GMM and the magnetic field intensity.

The strain variable of the GMM rod increases with the increase in magnetic field intensity and is independent of the magnetic field direction [32]. The length of the exciting winding should be larger than the GMM rod, so that the exciting winding is completely covered in its axial direction, improving the uniformity of the magnetic field and avoiding the edge effect. When selecting the range of working magnetic field intensity, this paper selects $AB/A_{1}B_{1}$ segments with linear changes according to the relationship between GMM strain and magnetic field intensity as shown in Figure 2, so as to facilitate the control of mechanical movement of the GMM rod. The minus sign of the abscissa in Figure 2 indicates the direction of the magnetic field.

#### 2.2.2. Determine the Bias Magnetic Field

**Figure 3:**
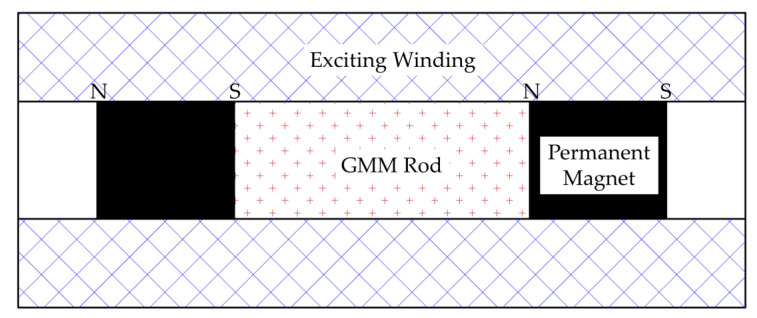
Disk type permanent magnet structure. “N” and “S” stand for magnetic poles.

Similar to the exciting winding selection, the GMM rod works well linearly in the bias field range of 15.9 kA/m to 39.8 kA/m, and the hysteresis is relatively small. After prestressing and materials are selected, the bias magnetic field is selected as the middle part of the linear region. In this paper, the bias magnetic field is the most common disc-type permanent magnet, which has a simple structure and does not exhibit a heating phenomenon like the DC type, as shown in Figure 3. However, due to the low permeability of GMM, magnetic leakage phenomena are likely to occur [33], so the magnetic field distribution of GMM rod is not uniform.

**Figure 4:**
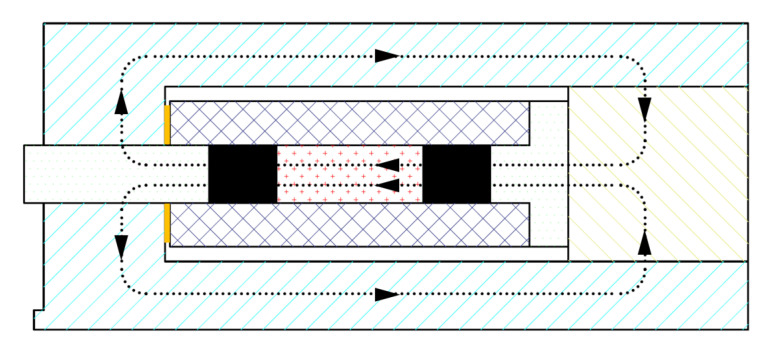
Direction of magnetic circuit.

In order to avoid magnetic leakage, the GMM rod is placed in the closed magnetic circuit for use in this paper. Air gaps should be avoided as much as possible in the construction of magnetic circuits. The closed magnetic circuit is composed of an outer shell, a GMM rod, exciting windings, drive elements, and other components, and the outer shell and drive element are all made of ferromagnetic materials to prevent magnetic leakage. The direction of the magnetic circuit is shown in Figure 4.

#### 2.2.3. Work Panel Design

The work panel is one of the core components of the transducer. Its shape, material, and constraint conditions will affect the output power, output force, vibration speed, and other characteristics of the transducer. Unreasonable design may reduce the efficiency of the transducer.

The resonant frequency of circular panel bending vibration [34] is:

(1) $$f=\frac{R^{2}\left(n\right)d}{2\pi r^{2}}\sqrt{\frac{E}{\rho \left(1-\mu^{2}\right)}}$$

where, ρ, μ, E, d, and r are the density, Poisson’s ratio, elastic modulus, thickness, and radius of the work panel, respectively; $R\left(n\right)$ represents the root of the lower-order frequency equation.

Based on the above analysis, the partial dimensions of the original configuration of the transducer designed in this paper are shown in Table 1.

## 3. FEM Simulation Analysis

### 3.1. FEM Theory of GMM

The finite element method is commonly used in the design of transducers. As a popular finite element analysis software at present, ANSYS has very comprehensive functions, but there is no module that can directly solve the electromechanical coupling problem of magnetostrictive materials. The piezoelectric effect is a force–electric conversion effect. When the piezoelectric ceramic is subjected to the excitation electric field, it will produce deformation. The magnetostrictive effect is similar to this. When subjected to the excitation current, the GMM rod has deformations such as elongation and shortening. It follows that the physical mechanism of the two effects is the same. This paper uses the piezoelectric–magnetoelectric analogy method [35,36]. The electromechanical coupling problem of magnetostrictive materials is analyzed by using ANSYS’s module that can solve the function of piezoelectric coupling.

In the finite element analysis, the piezoelectric equation of the piezoelectric coupling field [35] is:

(2) $$\left\{\begin{array}{c} T=c^{E}S-eE\\ D=eS+\varepsilon^{S}E \end{array}\right.$$

With such a ratio, the magnetoelectric equation can be obtained as:

(3) $$\left\{\begin{array}{c} T=c^{H}S-\beta H\\ B=\beta S+\mu^{S}H \end{array}\right.$$

where: T is stress, $c^{E}$ is elastic coefficient under constant electric field strength, S is strain, e is piezoelectric stress constant, E is electric field strength, D is electrical displacement, $\varepsilon^{S}$ is dielectric constant under constant strain, $c^{H}$ is elastic coefficient under constant magnetic field strength, β is magnetostrictive strain constant, H is magnetic field strength, B is magnetic induction strength, $\mu^{S}$ is permeability under constant strain.

According to (2) and (3), it can be obtained that:

(4) $$\mathrm{the}\;\mathrm{electroelastic}\text{–}\mathrm{magnetoelastic}\;\mathrm{equivalent}\;\mathrm{is}\;\left\{\begin{array}{c} c^{H}\Leftrightarrow c^{E}\\ \beta \Leftrightarrow e\\ \mu^{S}\Leftrightarrow \varepsilon^{S} \end{array}\right.$$

(5) $$\mathrm{and}\;\mathrm{the}\;\mathrm{electromagnetic}\;\mathrm{equivalent}\;\mathrm{is}\;\left\{\begin{array}{c} B\Leftrightarrow D\\ H\Leftrightarrow E \end{array}\right.$$

Using a generalized matrix and generalized vector, the finite element governing equation of the piezoelectric coupling problem [36] can be expressed as:

(6) $$\;\left[\begin{array}{cc} \left[M\right] & \left[0\right]\\ \left[0\right] & \left[0\right] \end{array}\right]\left[\begin{array}{c} \left[\ddot{u}\right]\\ \left[\ddot{V}\right] \end{array}\right]+\left[\begin{array}{cc} \left[C\right] & \left[0\right]\\ \left[0\right] & \left[0\right] \end{array}\right]\left[\begin{array}{c} \left[\dot{u}\right]\\ \left[\dot{V}\right] \end{array}\right]+\left[\begin{array}{cc} \left[K\right] & \left[K^{Z}\right]\\ {\left[K^{Z}\right]}^{T} & \left[K^{d}\right] \end{array}\right]\left[\begin{array}{c} \left[u\right]\\ \left[V\right] \end{array}\right]=\left[\begin{array}{c} \left[F\right]\\ \left[Q\right] \end{array}\right]$$

By analogy, the finite element governing equation of the magnetoelectric coupling problem can be written as:

(7) $$\;\left[\begin{array}{cc} \left[M\right] & \left[0\right]\\ \left[0\right] & \left[0\right] \end{array}\right]\left[\begin{array}{c} \left[\ddot{u}\right]\\ \left[\ddot{A}\right] \end{array}\right]+\left[\begin{array}{cc} \left[C\right] & \left[0\right]\\ \left[0\right] & \left[0\right] \end{array}\right]\left[\begin{array}{c} \left[\dot{u}\right]\\ \left[\dot{A}\right] \end{array}\right]+\left[\begin{array}{cc} \left[K\right] & \left[K_{m}\right]\\ {\left[K_{m}\right]}^{T} & \left[K_{\mu }\right] \end{array}\right]\left[\begin{array}{c} \left[u\right]\\ \left[A\right] \end{array}\right]=\left[\begin{array}{c} \left[F\right]\\ \left[\Phi \right] \end{array}\right]$$

where: $\left[M\right]$ is the mass matrix, $\left[u\right]$ is the structural displacement vector, $\left[V\right]$ is the potential vector at the joints of the piezoelectric body, $\left[C\right]$ is the damping matrix, $\left[K\right]$ is the stiffness matrix of the structure, $\left[K^{Z}\right]$ is the electromechanical coupling matrix, $\left[K^{d}\right]$ is the dielectric constant matrix, $\left[F\right]$ is the load force vector, $\left[Q\right]$ is the free charge accumulated on the cross section, $\left[A\right]$ is the number of excitation ampere-turns applied on the GMM, $\left[K_{m}\right]$ is the magnetostrictive coupling matrix, $\left[K_{\mu }\right]$ is the permeability matrix, $\left[\Phi \right]$ is the magnetic flux through the section.

### 3.2. Finite Element Modeling

**Figure 5:**
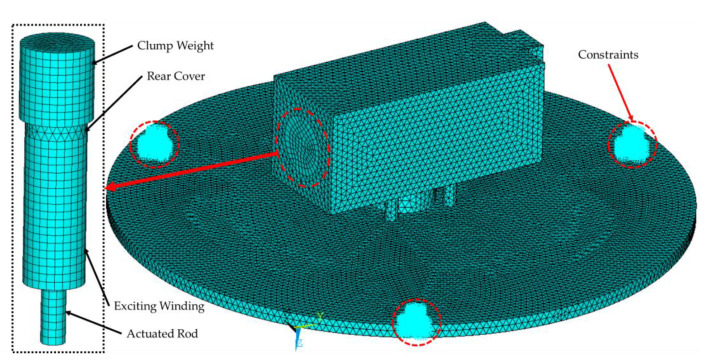
Grid and impose constraints.

In this paper, the finite element analysis software ANSYS (Canonsburh, PA, USA) is used for modal analysis of the designed transducer. Since the factors that affect the modal frequency of the transducer are mainly the work panel and the axial parts such as the GMM rod. Considering that the work panel will have drilled holes (the hole size is small), in the modeling process, to facilitate the subsequent addition of constraints, the grid division in small size and large number is adopted in the modeling to ensure the simulation’s accuracy. The grid division of the transducer is shown in Figure 5.

The material parameters used in the finite element simulation are shown in Table 2.

The SOLID186 structural quality unit was adopted to establish the model, and corresponding material properties were set for the axial structure, as shown in Table 3. The material of the drive element and rear cover was set to iron in order to form a good closed magnetic circuit and avoid magnetic leakage. The other parts of the transducer are made of steel.

**Figure 6:**
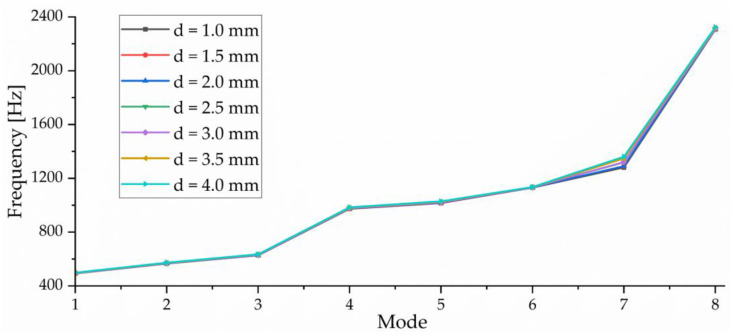
Mesh convergence study.

In order to ensure the calculation accuracy of the FEM, this paper studies the influence of mesh size on resonance frequency, as shown in Figure 6. When the mesh size d > 2.0 mm, the deviation of the resonance frequency obtained by numerical calculation increases obviously, especially for higher-order modes. However, when d = 1.0 mm, the number of grids exceeds one million, which is not conducive to improving computational efficiency. When d = 4.0 mm, the number of grids is only 20,000, but the calculation accuracy is too poor. Under the condition of ensuring the calculation accuracy, the grid size d = 2 mm is selected in order to improve the calculation efficiency. At this time, the number of grids is only 202,545, but the resonance frequency deviation obtained by calculation is not large.

During the simulation, the three screw holes on the work panel were constrained by the full degree of freedom, as shown in Figure 5. The LANB method was adopted to set the frequency solution range from 200 to 3600 Hz, and resonance frequencies of each order were obtained as shown in Table 4. Due to the limitations of the test equipment, the modal test may not be able to obtain all modal results. Therefore, in order to better compare with the modal test results and considering the higher power that can be obtained from the first mode [37], 11 modes are investigated in Table 4.

### 3.3. FEM Validation

**Figure 7:**
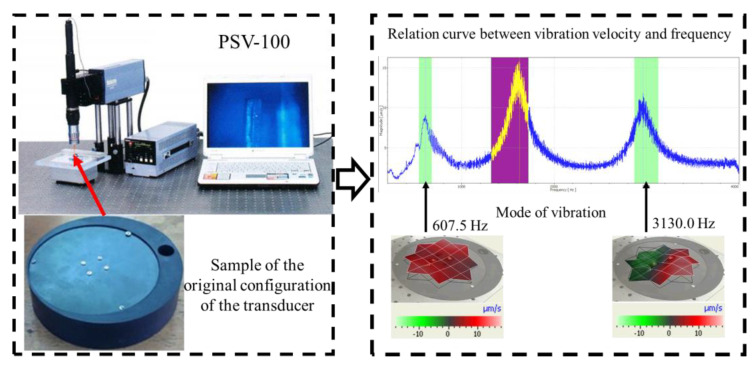
Grid and impose constraints.

In order to verify the accuracy of the finite element simulation results, a sample of the original configuration of the transducer is produced in this paper, and the PSV-100 laser vibration meter (Polytec GmbH, Karlsruhe, Germany) was used for test verification, as shown in Figure 7. The transducer, as a whole, is treated with rust prevention, and the sealing gasket is added to the axial component to avoid debris entering the exciting winding of the axial component and making it work abnormally. The transducer is only 40 mm thick.

**Figure 8:**
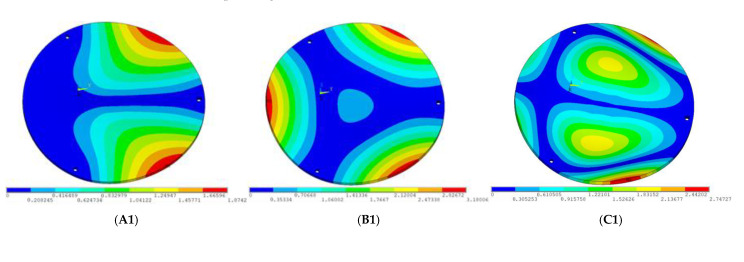

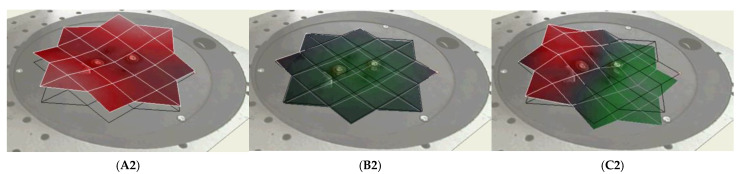
Comparison of simulation results and test results. (**A1**) *f*_1_ = 629.15 Hz. (**B1**) *f*_2_ = 1131.9 Hz. (**C1**) *f*_3_ = 3530.6 Hz. (**A2**) *f*_1_ = 607.5 Hz. (**B2**) *f*_2_ = 1321.3 Hz. (**C2**) *f*_3_ = 3130.0 Hz.

During the test, the same constraint conditions as the simulation were maintained, and the transducer’s resonant frequency within 200~4000 Hz was measured. The modal simulation results of the work panel were compared with the test results, as shown in Figure 8. In Figure 8A2,B2,C2 are the modal test results, while A1, B1, and C1 are the modes and resonance frequencies obtained from the finite element simulation corresponding to the modal test results.

It can be found that the obtained modes are basically the same, but the frequencies of each vibration mode are slightly different. In particular, the resonance frequencies of B1 and B2 and C1 and C2 are more different, while the resonance frequencies of A1 and A2 are relatively close. The reasons are summarized as follows:

(1) Due to the limitation of the test instrument, all the surface of the work panel is not divided into grids for the post-test, and only the part on the opposite side, namely the rectangular part, is tested. As a result, the number of measurement points is not enough, resulting in test errors. At the same time, some modes obtained in the simulation analysis may not be reflected in the test results of the modal test.
(2) In the modal test, the sample needs to constrain the boundary, so it is fixed to the rubber shell, but the rubber shell is not added in the simulation, resulting in a large error.
(3) The material parameters of simulation and test cannot be completely consistent, and the process defects of the sample during processing will also affect the results, and the difference between these parameters has a greater impact on the higher order modes.
(4) The error of the finite element simulation results will increase when calculating the higher-order modes [38].

## 4. Work Panel Optimization

### 4.1. Comparison of Basic Configurations

#### 4.1.1. Resonance Frequency Analysis

**Figure 9:**
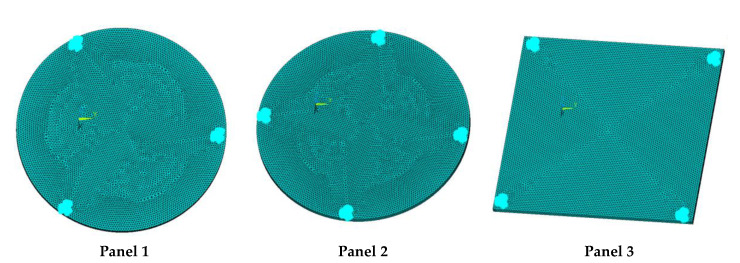
The three basic configurations of the work panel.

In order to lower the working frequency range of the designed transducer, three basic configurations of the work panel are proposed in this section (as shown in Figure 9), and they are simulated and compared. Among them, the area of the square disk and the circular disk is consistent. The constraints of the work panel can greatly affect the resonant frequency of the transducer. Therefore, Panel 1 and Panel 2 show two different constraints of the circular work panel for comparison, in which Panel 1 restricts three holes and Panel 2 restricts four holes. While Panel 3’s purpose is to investigate the influence of different shapes of work panels on resonance frequency.

Meanwhile, this paper only studies the vibration performance of the transducer below 2000 Hz, so only the first eight modes of the transducer are solved, and the modes and resonant frequencies of the three basic configurations are obtained as shown in Table 5.

Compared with Panel 1 and Panel 2, the resonance frequencies of most modes increase due to the increase in constraints, while Panel 2 and Panel 3 have the same constraints, but due to the change in shape of Panel 3, its resonance frequency decreases compared with Panel 2, and the lowest resonance frequency decreases most obviously. The lowest resonant frequency of Panel 3 is lower than that of Panel 1. Through comparison, it can be seen that the first-order resonance frequency of Panel 3 is the lowest, which is 24.6% lower than that of Panel 1. The highest resonant frequency of Panel 3 within 2000 Hz is 1744.9 Hz, which is 54.2% higher than that of Panel 1. Panel 3 has a smaller lowest resonance frequency, indicating that it can operate at lower frequencies. The mode of the work panel can affect the output power of the transducer in the frequency range of this mode. The frequency range studied in this paper is 0~2000 Hz. The highest resonance frequency of Panel 3 within 2000 Hz is higher, which can improve the working frequency bandwidth of the transducer and enable it to achieve high power output over a wider frequency range. In this way, the deformation of the work panel is also large, which greatly improves the working frequency range of the work panel. By comparison in terms of resonance frequency, Panel 3 is the best among the three basic configurations.

#### 4.1.2. Vibration Velocity and Sound Source Level Analysis

The working frequency range and vibration power of the transducer cannot be determined only by calculating the resonant frequencies of each mode. Therefore, this section simulates the acoustic vibration coupling of three basic configurations based on the harmonic response and harmonic acoustics modules of ANSYS. Then, the surface vibration velocity and sound source level (SL, reference sound pressure is $P_{0}=20\;\mu Pa$) of the three basic configurations are compared so as to determine the configuration of the work panel that can obtain a larger vibration power and working frequency range.

The resonant frequencies of mode 8 of the three basic configurations are all above and close to 2000 Hz, so mode 8 will affect the vibration power within 2000 Hz. Here, the simulation frequency range is set to 0~2400 Hz to ensure calculation accuracy within the range of 0~2000 Hz. In addition, the boundary of the computing domain is set as the radiation boundary to reduce the size of the computing domain and improve computing efficiency.

Meanwhile, 1 N of force is applied to the work panel during the simulation to simulate the force exerted by the drive element on the work panel. Since the area of the three basic configurations is equal, the influence of the applied force on the output power of the transducer can be excluded.

**Figure 10:**
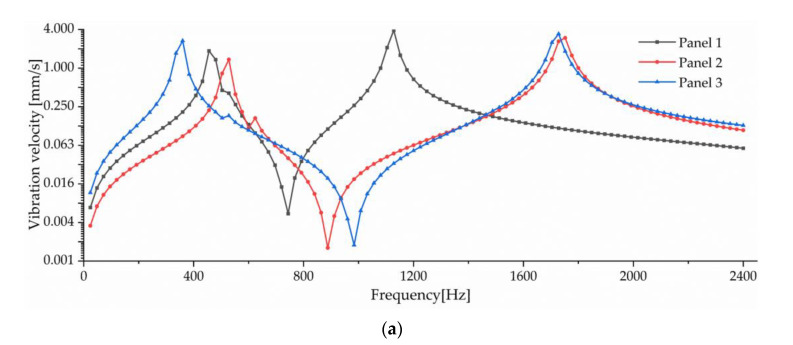

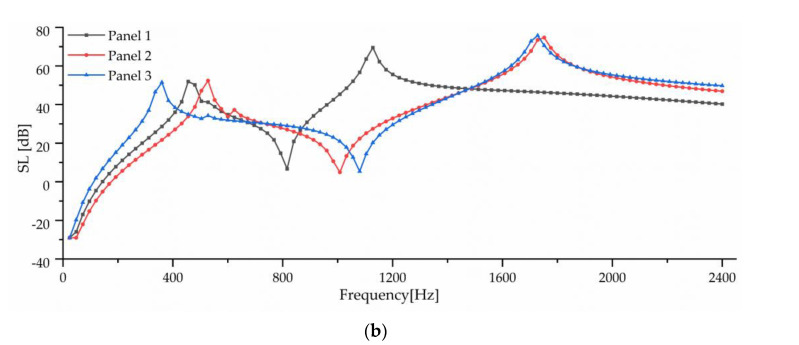
Comparison of vibration velocity and sound source level for three basic configurations. (**a**) Surface vibration velocity. (**b**) SL.

The surface vibration velocity and sound source level of the three basic configurations are shown in Figure 10. Among them, Panel 1 has a higher surface vibration speed and sound source level in the range of 900~1500 Hz, while Panel 3 also has a good vibration speed and sound source level in other frequency ranges. According to the requirement of a different working frequency range, the appropriate basic configuration can be selected as the work panel of the transducer.

Considering the frequency range of the study in this paper is 0~2000 Hz, combined with resonance frequency, surface vibration velocity, and sound source level analysis, it can be determined that Panel 3 is an optimal work panel configuration and can be applied to high-power sensors.

### 4.2. Honeycomb Panel Design

#### 4.2.1. Performance Analysis of Honeycomb Panels

**Figure 11:**
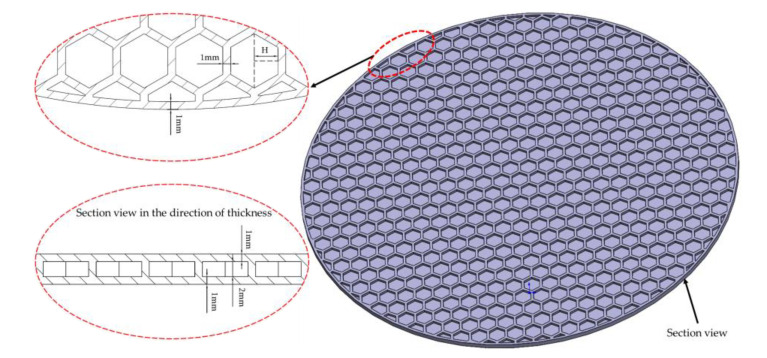
Structural parameters of the honeycomb panel.

The hexagonal cavity inside the honeycomb panel can significantly improve the vibration power of the work panel [39,40]. In this section, the work panel is innovatively designed as a honeycomb panel structure (as shown in Figure 11, where half opposite sides of a hexagon H = 3 mm) to study the influence of honeycomb structure on the vibration performance of the work panel. At this time, due to the influence of the honeycomb wall thickness, this paper changes the grid size of the work panel to 1 mm to ensure the calculation’s accuracy.

**Figure 12:**
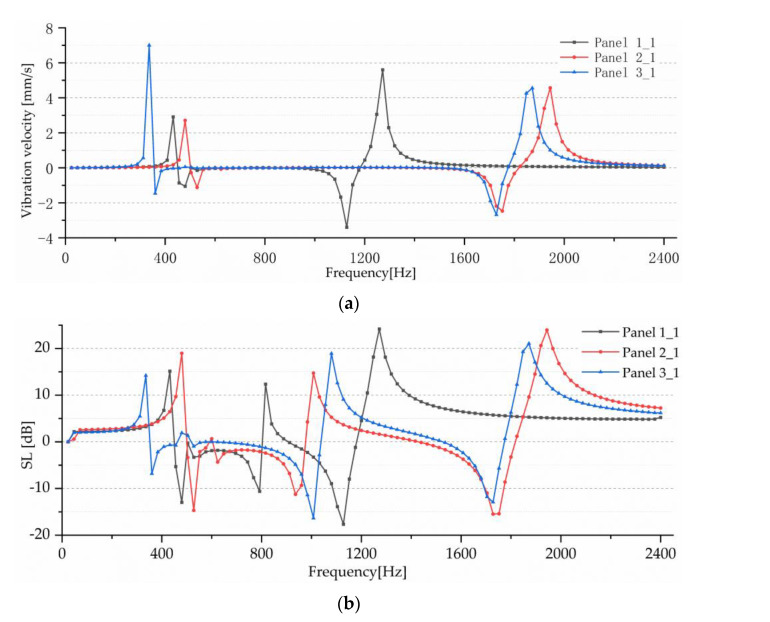
Simulation results of honeycomb panels. (**a**) Surface vibration velocity. (**b**) SL.

The simulation analysis results are shown in Figure 12. Panel 1_1, Panel 2_1, and Panel 3_1 are the incremental curves of surface vibration velocity and sound source level obtained after Panel 1, Panel 2, and Panel 3 were modified into honeycomb panels, respectively. By comparison, it can be seen that the design of the honeycomb panel can significantly improve the vibration power of the work panel, but the power of the honeycomb panel significantly decreases within a certain frequency range (such as 1100~1150 Hz). Through analysis, it can be seen that the resonance frequency of the panel changes slightly due to the quality reduction of the honeycomb panel and the internal vibration of the honeycomb structure. In general, the design of the honeycomb panel has a very ideal effect on improving the vibration power of the work panel, thus verifying the superiority of the honeycomb structure.

#### 4.2.2. Optimization of Honeycomb Structure Parameters

**Figure 13:**
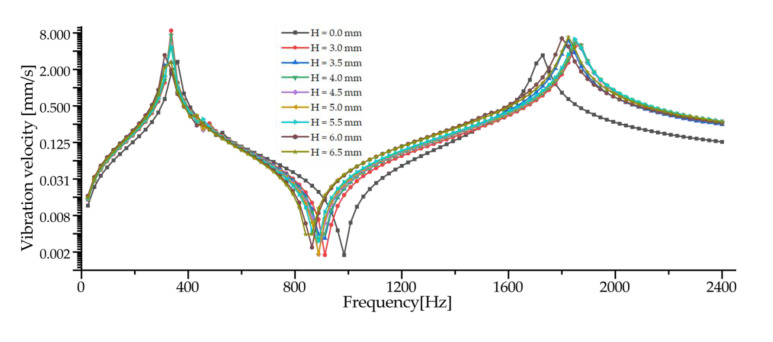

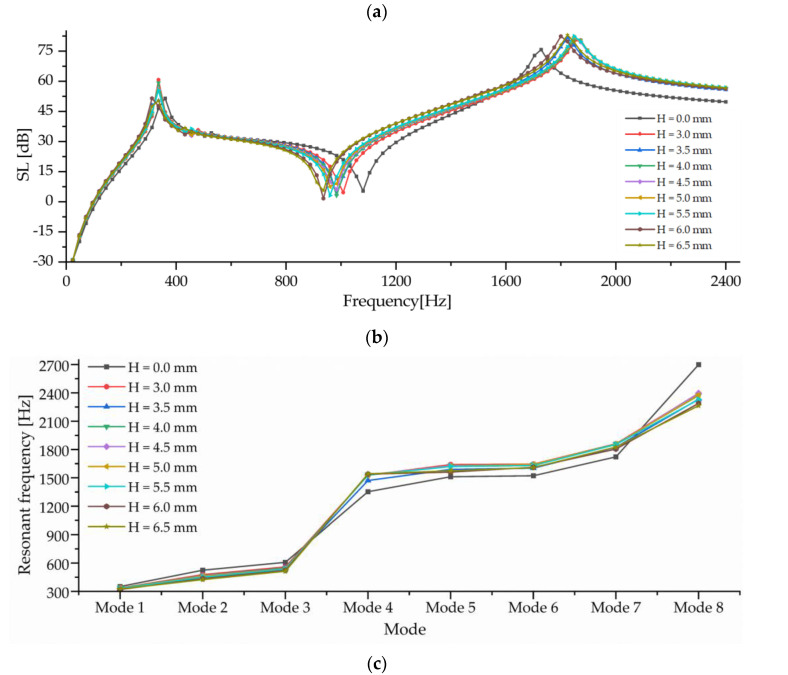
The influence of value H on the work panel. (**a**) Surface vibration velocity. (**b**) SL. (**c**) Resonance frequency.

The design of the honeycomb panel can significantly improve the vibration power of the work panel. In this section, Panel 3 with better vibration performance is taken as the basic configuration of the study, and the influence of honeycomb size on vibration power and resonance frequency is explored by changing the distance H of the half opposite sides of a hexagon in the honeycomb structure, as shown in Figure 13.

According to the analysis of Figure 13a,b, the vibration power of the work panel increases in the range of 0~700 Hz, but there is no linear relationship with the value of H, and the power is larger when H = 3.0~4.0 mm. Compared with Panel 3 (H = 0 mm), the vibration power in the range of 700~900 Hz is decreased, but the decrease of H = 3.5~5.5 mm is smaller. By comparison with Figure 13c, it can be seen that the mode 3 resonance frequency of the honeycomb panel decreases, while the mode 4 resonance frequency increases, resulting in the vibration of the work panel within the frequency range being less affected by the resonance, thus reducing the vibration power. In the range of 900~2000 Hz, the similar honeycomb structure greatly improves the vibration power. It can be seen that the design of the similar honeycomb panel has a great influence on the vibration performance at high frequencies, and the amplitude of the vibration power of H = 3.5 mm and H = 6.0~6.5 mm is higher.

The analysis of Figure 13c shows that the design of the honeycomb panel can reduce the frequencies of the first three vibration modes, but the frequency of mode 4~7 is increased, but the increase in resonance frequency is small. At the same time, the design of the honeycomb panel can significantly reduce the vibration frequency of mode 8, resulting in a significant increase in the influence of mode 8 on the vibration power of 0~2000 Hz. Generally speaking, the design of honeycomb panels can improve the vibration frequency of work panels, and the effect is better when H = 3.5 mm. Compared with Panel 3, the lowest resonant frequency of H = 3.5 mm is 324.71 Hz, reduced by 12.8%.

By synthesizing Figure 13a–c, it can be concluded that the honeycomb panel with H = 3.5 mm is a better design for the work panel, which realizes the optimization of the work panel.

The honeycomb panels have been commercialized, and the difficulty of processing honeycomb panels has been greatly reduced. At the same time, the size of the honeycomb panel designed in this paper is similar to that of existing commercial products, and even the accuracy requirement is lower than some honeycomb panels sold in the market. Therefore, the processing difficulty of the honeycomb panel is slightly higher than that of the conventional panel, but not much higher. Therefore, the honeycomb panel designed in this paper can be realized by the existing processing technology. At the same time, the design of honeycomb board also realizes the weight reduction of the transducer, which has far-reaching significance for aerospace.

## 5. Conclusions

Based on GMM, this paper proposes a lightweight transducer suitable for low-frequency applications and oriented to the aerospace industry, aiming to explore ways to solve the problems such as low vibration power and the short working bandwidth of low-frequency sensor. The working principle of the transducer is introduced in detail, and the design process of the core parts such as the exciting winding, the bias magnetic field, and the work panel is emphatically expounded. The dimensions of the structure of each part are given. The numerical simulation results are verified by the laser vibration meter, and the structure of the work panel is optimized. The detailed conclusions are as follows:

(1) The original configuration of the GMM transducer is proposed. The actual working conditions and material properties of each part are considered in the finite element modeling and numerical simulation, and the effectiveness of the finite element simulation is verified by comparing it with the modal test. The original configuration preliminarily realizes the functions of low frequency, high efficiency, light weight, and high power, which provides a reference for the subsequent structural optimization.
(2) Three basic configurations of the work panel are proposed and developed, and the optimal panel, Panel 3, is selected as the configuration of the work panel through modal, acoustic, and vibration coupling analysis and comparison. The resonant frequency of mode 1 of Panel 3 is 372.47 Hz, which is 24.6% lower than the original configuration. At the same time, the highest resonant frequency of Panel 3 within 2000 Hz is 1744.9 Hz, which greatly improves the working bandwidth and vibration power of the panel.
(3) The honeycomb structure is innovatively applied to the work panel, which verifies that the honeycomb structure has a good effect on the vibration power of the work panel. The dimensional parameters of the honeycomb structure are optimized by considering the surface vibration velocity, sound source level, and resonance frequency. It is obtained that the honeycomb structure with the half opposite sides of a hexagon (H = 3.5 mm) has the highest performance improvement on the work panel. Comparing with Panel 3, the lowest resonance frequency was reduced by 12.8%.

The thin transducer designed in this paper based on GMM can realize low-frequency and high-power operation below 2000 Hz through structural optimization design and has the characteristics of small size, light weight, large broadband, high efficiency, and high power. It has a certain engineering application prospect in the field of low frequency broadband transducers and provides a new idea for the aerospace sensor to realize low-frequency, large broadband and high-power operation.

## Figures and Tables

**Table 1:** Structural dimension parameters.

Structure Name	Size/mm
work panel	ϕ170×4
GMM rod	ϕ6×15
permanent magnet	ϕ6×7
clump weight	ϕ18×18.5
exciting winding	$\phi 15\left(\mathrm{outside}\;\mathrm{diameter}\right)/\varphi 6\left(\mathrm{inside}\;\mathrm{diameter}\right)\times 37$
outer shell	section side length: 31.5, axial length: 72.5

**Table 2:** Material parameters.

Materials	Elasticity Modulus *E*/GPa	Density *ρ*/kg·m^−3^	Poisson’s Ratio *μ*
steel	209	7890	0.269
GMM	23	9250	0.210
iron	194	7930	0.280
copper	90	8500	0.300
magnet	113	3800	0.230

**Table 3:** Corresponding table between structures and materials.

**Structures**	clump weight	actuated rod	permanent magnet	GMM rod	exciting winding	rear cover
**Materials**	steel	iron	magnet	GMM	copper	iron

**Table 4:** The simulated resonance frequency of the original configuration.

**Mode**	1	2	3	4	5	6	7	8	9	10	11
**Frequency *f*/Hz**	494.06	567.56	629.15	975.65	1018.7	1131.9	2310.0	2441.0	2448.0	2758.7	3530.6

**Table 5:** The modes and vibration frequencies of three basic configurations.

Resonant Mode	Panel 1	Panel 2	Panel 3
mode 1	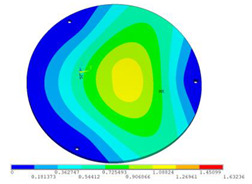	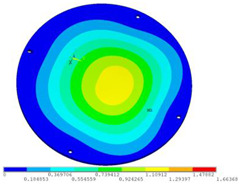	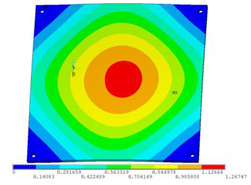
	494.06 Hz	551.41 Hz	372.47 Hz
mode 2	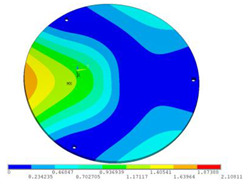	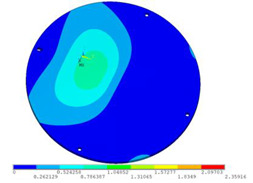	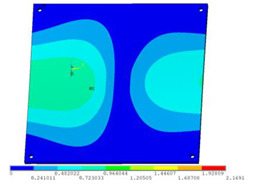
	567.56 Hz	651.86 Hz	563.60 Hz
mode 3	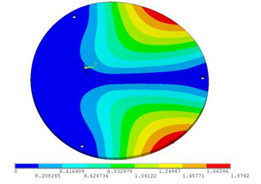	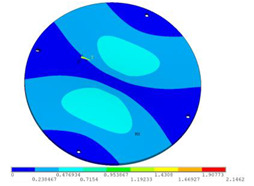	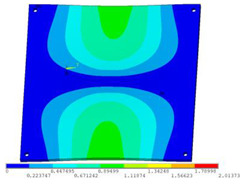
	629.15 Hz	744.26 Hz	645.18 Hz
mode 4	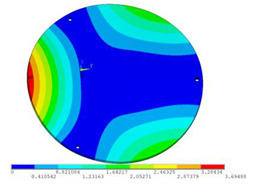	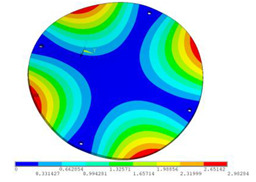	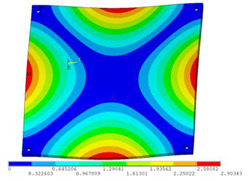
	975.65 Hz	1380.0 Hz	1352.1 Hz
mode 5	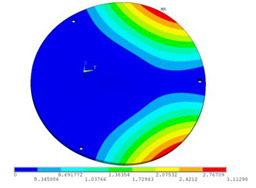	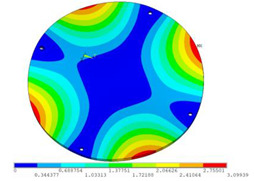	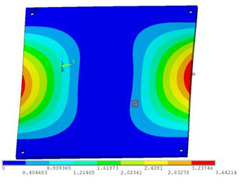
	1018.7 Hz	1566.4 Hz	1521.7 Hz
mode 6	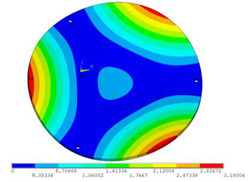	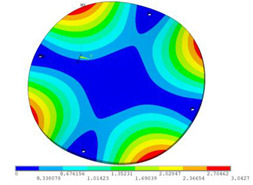	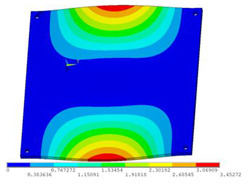
	1131.9 Hz	1572.2 Hz	1540.9 Hz
mode 7	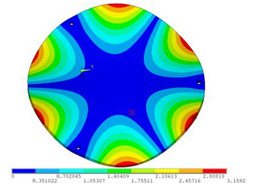	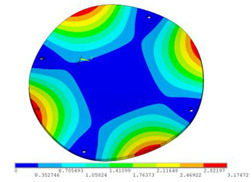	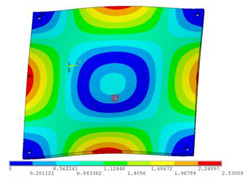
	2310.0 Hz	1745.3 Hz	1744.9 Hz
mode 8	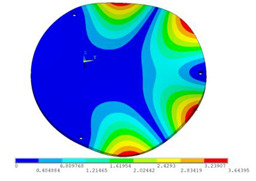	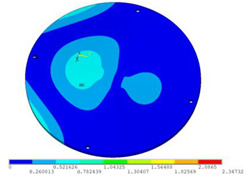	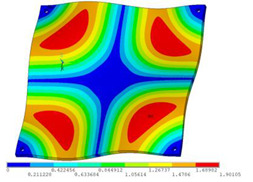
	2441.0 Hz	2776.5 Hz	2705.1 Hz

## Data Availability

The data presented in this study are available on request from the corresponding author.
